# Relating goal-directed behaviour to grazing in persons with obesity with and without eating disorder features

**DOI:** 10.1186/s40337-020-00324-1

**Published:** 2020-10-01

**Authors:** Andreea I. Heriseanu, Phillipa Hay, Laura Corbit, Stephen Touyz

**Affiliations:** 1 Clinical Psychology Unit, School of Psychology, University of Sydney, Level 3, Building M02F, 94 Mallett St, Camperdown, NSW 2050 Australia; 2 eCentreClinic, Department of Psychology, Macquarie University, Macquarie Park, NSW 2109 Australia; 3 Translational Health Research Institute, School of Medicine, Western Sydney University, Locked Bag 1797, Penrith, NSW 2751 Australia; 4 Campbelltown Hospital, South West Sydney Local Health District (SWSLHD), PO Box 149, Campbelltown, NSW 2560 Australia; 5 Department of Psychology, University of Toronto, 100 St. George Street, Toronto, ON M5S 3G3 Canada; 6 Inside Out Institute, Charles Perkins Centre, University of Sydney, Johns Hopkins Drive, Camperdown, NSW 2006 Australia

**Keywords:** Grazing, Obesity, Eating disorders, Goal-directed behaviour, Habit, Compulsive eating, Decision-making

## Abstract

**Background:**

Both obesity and eating disorders (ED) have been associated with reductions in purposeful, flexible goal-directed behaviour, and with an overreliance on more rigid habitual behaviour. It is currently unknown whether grazing, an eating style which is common in both conditions, is related to goal-directed behaviour. The current study therefore aimed to relate grazing to goal-directed behaviour in a group of participants with obesity with and without ED features, compared to a healthy-weight control group.

**Methods:**

Participants (*N* = 87; 67.8% women, mean age 28.57 years), of whom 19 had obesity and significant eating disorder features, 25 had obesity but without marked eating disorder features, and 43 were age- and sex-matched healthy-weight controls, completed two instrumental learning tasks assessing action-outcome contingency sensitivity and devaluation sensitivity, as well as demographic and eating disorder-related questionnaires. Gamma and Ordinary Least Squares regressions were performed to examine the effect of group and grazing on goal-directed behaviour.

**Results:**

Lower action-outcome contingency sensitivity was found in the group with obesity and with eating disorder features than in the group with obesity but without eating disorder features or in healthy controls. No group differences in devaluation sensitivity were found. A small but significant relationship was found between grazing severity and contingency sensitivity in the group with obesity and eating disorder features, such that increasing grazing severity was associated with less diminished contingency sensitivity.

**Conclusions:**

There is some indication that in persons with obesity and eating disorder features instrumental behaviour is less flexible and adaptive; furthermore, within this group grazing may represent a goal-directed behaviour, despite unhelpful long-term implications of grazing.

## Plain English summary

People with high weight and/or eating disorders can act in ways that are habitual and rigid. Grazing is the repetitive and unplanned eating of small amounts of food that sometimes includes a feeling of loss of control, i.e. that you cannot stop or resist eating. Grazing is common in eating disorders and obesity, but it is unknown if grazing is related to acting in a more habitual or rigid way. This study aimed to look at this potential connection in three groups: a group of people with high weight and symptoms of eating disorders, a group with high weight but without eating disorder symptoms, and a group with normal weight without eating disorder symptoms, by using a computer program resembling a food vending machine. Those with high weight and eating disorder symptoms were less likely than the other groups to change their behaviour to maximize obtaining a picture reward, despite the fact that they had knowledge of how to do this. In this group, those with more severe grazing were slightly better at updating their behaviour, which may show that in this group grazing is connected to behaviour that is more flexible.

## Background

The effects of obesity are recognised as a leading global health concern [1], however, its causes and maintaining factors are complex and not well understood, involving a range of genetic, epigenetic, social, environmental, physiological and psychological factors [2]. For many individuals, gaining weight is hard to prevent and difficult to reverse. An additional challenge for persons with higher weight is the co-occurrence of eating disorders (ED), especially binge eating disorder [3, 4]. Where obesity and eating disorders co-occur, in addition to increases in disordered eating cognitions and behaviours, there are less favorable outcomes for individuals undergoing weight loss treatment such as bariatric surgery, as well as increased risk of mood and anxiety disorders, and reduced quality of life and psychosocial functioning [5]. Recent epidemiological research has indicated that rates of disordered eating within obesity have had a higher increase in prevalence over the past decade than either obesity or disordered eating alone [6].

Health behaviour change is often a treatment focus in groups with higher weight. One of the barriers to long-term change in frequently-targeted behaviours (such as dietary patterns and physical inactivity) is that these behaviours are often habitual and operate largely outside of conscious awareness once established [7, 8]. Studies in both humans and rodents have found evidence that instrumental behaviour (defined as voluntary behaviour, acting upon the environment to elicit a response [9]) is governed by two dissociable systems: an action-outcome goal-directed system which is present in early stages of learning, and a stimulus-response habitual system, which becomes more dominant with continued training [10]. Goal-directed behaviour is flexible and sensitive to both the action-outcome contingency (i.e. the causal relationship between an action and its consequence) and to the value of the outcome [11]; however, sustained volitional control over behaviour requires considerable cognitive effort [12]. Conversely, habitual behaviour operates without the necessity of deliberate intentions, and therefore is less cognitively demanding [13, 14]; however, with repetition, behaviour becomes more rigid, increasingly guided by triggering stimuli (such as cues in the environment) and independent of the value of the outcome, or of the action-outcome contingency [15, 16].

In instrumental decision-making tasks, obesity has been shown to be associated with both reduced goal-directed control over food choices [17] and with habit-like responding for snack food [18]. There is also some indication that stronger reductions in goal-directedness and/or shifts towards habitual behaviour may be present in those with obesity and comorbid ED [19]. Participants with obesity and binge eating disorder reported higher reward sensitivity than controls of similar weight [20]: this is the tendency to seek, learn from, and derive pleasure from positive stimuli [21], which affects individuals’ motivation to engage in goal-directed behaviour [22]. Reward sensitivity was also positively correlated with prefrontal cortex activation, suggesting changes in reward value representation, as the prefrontal cortex is thought to include representations of contingencies, and outcomes and their value [23]. Increased activation in the dorsal striatum, an area which increasingly influences behaviour as it shifts to becoming more habitual [24, 25], was also found in participants with obesity and binge eating compared to a non-binge eating group with obesity during a food stimulation task [26]. The results regarding decision-making in those with obesity and ED are not unequivocal, however; although a recent meta-analysis found evidence for disadvantageous reward-related decision-making in human adults with obesity as well as in those with ED [27] (suggesting impairments in neurocognitive functioning in both groups), it did not find differences between participants with obesity with and without binge eating disorder.

While the neurobehavioural profile of binge eating is the most studied to date within both obesity and ED, eating patterns across both conditions are diverse [28] and other types of eating exist which also display compulsive dimensions [29]; therefore, a more inclusive range of eating patterns need to be considered in the context of research and clinical practice. Grazing is an eating pattern initially and predominantly examined within the bariatric surgery field [30]. However, recent examination has found it be frequently-occurring in community groups with obesity, ED, and at their overlap [31, 32]. Grazing has been defined by expert consensus as the unplanned, repetitive eating of small amounts of food, and/or eating not in response to hunger/satiety sensations [33], and has been conceptualised as existing on the spectrum of compulsive eating [34]. It represents a clinically significant form of overeating, especially when a sense of loss of control over grazing is a central feature, which is relatively common (in a recent study surveying a representative community sample, 26.9% of those who regularly grazed experienced loss of control when grazing [32]). Grazing is particularly prevalent in groups with higher weight and binge-type EDs [32]. Although a tendency to graze is strongly associated with a tendency to binge eat, they represent discrete eating patterns: grazing does not involve the consumption of food in a discrete period of time, it does not always incorporate a sense of loss of control; and amounts consumed are not always considered objectively or subjectively “large” [35]. Furthermore, a study found that the relationship between binge eating and grazing was not accounted for by a sense of loss of control [36], even though this is a core feature of binge eating, and is also relatively common in grazing. Associations have been found between compulsive grazing (i.e. grazing incorporating a sense of loss of control) and reduced treatment success for obesity [37, 38], psychological distress [36], and eating disorder symptoms [38]. A recent study has found associations between compulsive grazing and symptoms of food addiction [39], and a model of obesity maintenance (COMM [40];) suggests that atypical eating behaviours including grazing are influenced by habit strength, thus contributing to the cycle maintaining high weight. Given its unplanned, repetitive nature, and its occurrence outside of hunger/satiety signals, grazing (especially when high in compulsivity) can be theoretically linked to the stimulus-response/habitual action system, prompted by the availability of food-related cues in the environment [23], potentially resulting from failures of self-regulation.

Few studies on obesity-maintaining behaviours have formally incorporated the role of habit or goal-directed behaviour [17–19, 41], and there is also very little information on the neurobehavioural basis of decision-making involving grazing behaviour within obesity and/or ED. Moreover, research investigating the role of habit in health behaviours has traditionally used explicit self-report measures, rather than implicit instrumental tasks, despite the reflexive, automatic nature of habits [12], which often places these behaviours outside of conscious self-awareness. Considering the degree to which behaviour is goal-directed in the context of obesity with and without ED features may help elucidate some of the maintaining mechanisms - for example, if eating patterns and physical activity have a more habitual basis in these conditions, they may be driven by cues in the environment, and may be more resistant to shifting through traditional interventions such as providing information aimed at improving knowledge. Therefore, the current study aims to examine whether participants with obesity with and without ED features display impairments in goal-directed behaviour compared to each other and to a healthy-weight control group, and to relate the degree of goal-directed behaviour in these groups to grazing.

## Methods

### Participants

Participants recruited from community and university settings were screened via telephone prior to the experiment. Adult participants (aged between 18 and 65 years), whose BMI was either between 18.5 and 25 kg/m^2^ or over 30 kg/m^2^ and who had completed at least 10 years of education in English were enrolled. Exclusion criteria consisted of: a history of psychosis or mania, neurological injuries or disorders, learning disorders, hearing/visual impairment, regular sedative/ or stimulant use, substance use difficulties and current participation in weight loss treatment programs. Community participants received a $20 shopping card as reimbursement, while university students received course credit. The study was approved by the University of Sydney Human Research Ethics Committee (Approval No.: 2014/936), and written informed consent was obtained from all participants.

### Measures

#### Demographic and health data

A custom questionnaire was used to collect information on age, sex, residential postcode (used to estimate household income), ethnicity, country of birth, education, marital status, medical and psychiatric history, medication use, alcohol consumption, cigarette smoking, illicit substance use, exercise, and onset of obesity.

#### Anthropometric measurements

Height in metres and weight in kilograms were measured using Tanita Wedderburn BWB-700 scales and stadiometer, to calculate BMI (kg/m^2^). Participant height and weight were measured while wearing light clothing and no shoes.

#### Presence of marked ED features

Participants completed the Eating Disorder Examination-Questionnaire (EDE-Q [42];), a 28-item self-report questionnaire assessing ED psychopathology. The EDE-Q has been found by various studies to be a valid and reliable questionnaire [43]; in the current study, α = 0.95. Participants were classified as likely and not likely to have an ED based on the method derived by Mond et al. [44]: (1) EDE-Q Global Score ≥ 2.3 AND (2) the occurrence of any objective binge episodes OR excessive (driven/compulsive) exercising at least once per week. Compulsive exercise is considered to be a maladaptive compensatory behaviour, used as a means of controlling shape, weight, amount of fat, or to burn off calories [42].

#### Grazing severity

The Grazing Questionnaire (GQ [36];) is a seven-item self-report scale measuring the frequency of unplanned, continuous and repetitive eating of small amounts of food through extended time periods, with higher scores indicating higher severity. GQ items are rated on a 5-point scale, ranging from “0 – Never” to “4 – All of the time”. The GQ total score is calculated by the addition of the seven items; the GQ includes two factors: “repetitive eating” (four items) and perceived “loss of control” (three items). The GQ was found to possess high internal consistency, test-retest reliability and convergent validity in a sample of undergraduate psychology students [36] and in a mixed sample of students and community participants [45]; in the present study, α for the entire GQ was 0.89, for the “repetitive eating” factor α = 0.84, and for the “loss of control” factor, α = 0.88. The two factors were significantly and strongly positively correlated, *r* = 0.66, *p* < .001.

#### Snack food preference and pleasantness ratings

At two time points (T1: prior to task administration; and T2: following the conclusion of the two tasks) participants were asked to rate their liking for three snack food items (chocolates: M&Ms., sweet biscuits: Tiny Teddies, and savoury snacks: BBQ Shapes), with the following instruction: “Rate the pleasantness of BBQ Shapes/M&Ms/Tiny Teddies below”. This was rated on a seven-point scale, from 1 (“very unpleasant”) to 7 (“very pleasant”); see Additional File 1. For each participant, the two snacks that were closest in pleasantness were chosen as outcomes (presented pictorially) for the subsequent tasks.

#### Goal-directed behaviour

Two tasks (Contingency Variation Task: CVT; and Outcome Devaluation Task; ODT) were used to determine the extent to which instrumental behaviour was goal-directed. The tasks were developed by researchers at the Brain and Mind Research Institute, University of Sydney as human analogues for well-established animal research paradigms investigating instrumental behaviour [46]. The CVT aims to test whether behaviour can flexibly adapt to respond for a higher action-outcome contingency over a lower contingency, and the ODT aims to test whether behaviour is sensitive to the value of a reinforcer, or rigid and habitual and hence insensitive to an altered value of the reinforcer following devaluation. The tasks also test if participants can correctly identify reward contingencies and whether they experience subjective devaluation, and tests for dissociations between behaviour and knowledge of contingency/outcome value. A detailed description of these tasks can be found in Additional File 1. Presentation of stimuli and recording of button presses for both tasks was implemented using PsychoPy Version 1.81.03 [47], on a MacBook Air 11″ laptop computer using the OS X El Capitan operating system. Behavioural outcomes consisted of: Contingency Sensitivity Index (CSI) for the CVT, calculated as the number of keypresses performed for the high contingency outcome divided by the total number of presses; and the Devaluation Sensitivity Index (DSI) for the ODT, calculated as the number of extinction keypresses for the non-devalued outcome divided by the total number of extinction presses (similar to Dietrich [48]). Knowledge outcomes consisted of mean contingency ratings for the high contingency and the low contingency outcome for the CVT; and difference in pleasantness ratings between T1 and T2 for the devalued vs the non-devalued outcome for the ODT.

#### Depression severity

The Depression Anxiety Stress Scales-21 (DASS-21) [49] Depression subscale was used for assessing depression severity. This includes seven items measuring levels of self-reported depression over the past week. Responses are rated on a five-point Likert scale ranging from 0 (“Did not apply to me at all”), to 4 (“Applied to me very much, or most of the time”). Subscale scores represent the sum of responses, with higher scores indicating higher severity. The DASS-21 has been shown to have good-to-excellent internal consistency in clinical and community samples [50]; in the current study, α for the total scale was 0.85, and for the Depression subscale, α = 0.90.

#### Estimated overall intellectual functioning

Full-scale IQ was estimated using the Test of Premorbid Functioning (TOPF) [51]. This test consists of a list of 70 words with atypical grapheme to phoneme translations which participants are asked to read aloud. The raw score consists of the total number of words pronounced correctly, ranging from 0 to 70; this score was converted to a standard score using age norms.

#### Hunger level

Participants were not required to fast prior to the experiment; instead, they were asked to rate their hunger level on a Likert scale of 1 to 10, where a higher number represented a higher level of hunger.

### Procedure

Participants first completed a face-to-face assessment consisting of anthropometric measurement (height in metres and weight in kilograms), a self-report questionnaire containing demographics and measures of eating psychopathology and mood, followed by TOPF administration, T1 snack pleasantness ratings, and two computerised instrumental tasks (CVT and ODT). Finally, participants completed the T2 snack pleasantness rating.

### Statistical plan

Statistical analyses were performed using IBM SPSS Statistics version 26. The three groups (1. participants with obesity and significant ED symptoms; 2. participants with obesity but without significant ED symptoms; 3. healthy-weight controls without significant ED symptoms) were compared on demographic and clinical variables using ANOVAs (or Welch tests, if the homoscedasticity assumption was not met) for continuous variables, and χ^2^ tests for categorical variables. For all tests a two-tailed α of .05 was used, and for pairwise contrasts the Sidak correction was employed for continuous variables and the Bonferroni correction for categorical variables. For CSI and DSI, Gamma Regression was used through the Generalised Linear Models procedure. For differences in contingency ratings and in pleasantness ratings, Ordinary Least Squares Regression was used through the Generalised Estimating Equations procedure. Multicollinearity statistics were checked and were found to be within acceptable parameters. All analyses utilised heteroscedasticity-consistent robust standard errors. Unadjusted analyses were first conducted, followed by analyses adjusted for age, sex, years of education, estimated overall intellectual functioning and depression severity as recommended in prior cognitive function research [52]. Where between-group differences were found, the effect of grazing severity (entered as a continuous variable) was examined within each of the three groups, as these varied considerably in terms of grazing severity.

Estimates for power vs. sample size, calculated using the G*Power 3 software, indicated that with three groups, six covariates, α = .05, β = .80, and 87 participants, the power for detecting a large effect size (*f* = 0.40) is 0.80.

## Results

### General sample characteristics

A total of 90 participants recruited from community and university settings were enrolled in this study, but three were excluded, as detailed below. The final sample (*N* = 87) was 67.8% female, with a mean age of 28.57 years (SD = 8.70, range: 18.18–58.34 years), and with 16.47 years of education on average (SD = 2.42, range: 12.00–25.00 years); 54.0% were married or in a relationship, and 55.2% were born in Australia. Forty-three participants had a BMI between 18.5 and 25 kg/m^2^ and did not endorse marked ED features and were categorised as healthy controls (HC group); 25 participants had a BMI of at least 30 kg/m^2^ and did not endorse marked ED features (OB group), and 19 participants had a BMI of at least 30 kg/m^2^ and endorsed prominent ED psychopathology (OBED group). Demographic and clinical characteristics of the three groups can be found in Table 1. No significant between-group demographic differences were observed.

**Table 1:** Demographic and clinical characteristics

	HC (*n* = 43)	OB (*n* = 25)	OBED (*n* = 19)	*F/Welch*/χ^2^ statistic	Pairwise comparison
	*n%; M(SD)*	*n%; M(SD)*	*n%; M(SD)*		
Source of recruitment (community/university)	41 (95.3) / 2 (4.7)	21 (84.0) / 4 (16.0)	17 (89.5) / 2 (10.5)	2.68	–
Age (years)	27.81 (7.53)	30.17 (10.82)	28.20 (8.25)	0.60	–
Sex (female/male/other)	32 (74.4) / 11 (25.6) / 0 (0.0)	13 (52.0) / 12 (48.0) / 0 (0)	14 (73.7) / 5 (26.3) / 0 (0.0)	4.02	–
Ethnicity (Caucasian/Asian/other)	24 (55.8) / 17 (39.5) / 2 (4.7)	14 (56.0) / 10 (40.0) / 1 (4.0)	13 (68.4) / 4 (21.1) / 2 (10.5)	2.83	–
Income (AUD$1000)	61.56 (14.72)	57.29 (10.28)	61.71 (13.65)	0.93	–
Country of birth (Australia/other)	20 (46.5) / 23 (53.5)	13 (52.0) / 12 (48.0)	15 (78.9) / 4 (21.1)	5.75	–
Marital (married or in relationship)	28 (65.1)	9 (36.0)	10 (52.6)	5.42	–
Education (years)	16.80 (2.31)	16.80 (2.76)	15.29 (1.85)	3.84	–
BMI (kg/m^2^)	22.31 (2.07)	33.98 (3.11)	38.44 (5.73)	188.79***	OBED > OB > HC
Obesity onset (child/adolescent/adult)	–	6 (24.0) / 9 (36.0) / 10 (45.5)	2 (10.5) / 5 (26.3) / 12 (63.2)	2.55	–
Alcohol (std. drinks/week)	2.17 (3.21)	2.42 (3.10)	1.91 (3.23)	0.14	–
Smoking (never/past/current)	40 (93.0) / 1 (2.3) / 2 (4.7)	21 (84.0) / 0 (0.0) / 4 (16.0)	14 (73.7) / 1 (5.3) / 4 (21.1)	5.60	–
Cholesterol medication	0 (0.0)	1 (4.0)	0 (0.0)	2.51	–
Blood pressure medication	0 (0.0)	1 (4.0)	0 (0.0)	2.51	–
Antidepressant medication	1 (2.3)	1 (4.0)	3 (15.8)	4.61	–
Trying to lose weight	4 (9.3)	19 (76.0)	16 (84.2)	43.68***	OB/OBED > HC
Physical activity (< 1 h/1-5 h/> 5 h per week)	6 (14.0) / 24 (55.8) / 13 (30.2)	5 (20.0) / 17 (68.0) / 3 (12.0)	3 (15.8) / 14 (73.7) / 2 (10.5)	4.91	–
TOPF	111.33 (11.02)	110.48 (12.02)	111.47 (13.80)	0.05	–
EDE-Q Global Score	0.68 (0.65)	1.75 (0.82)	3.40 (0.72)	94.91***	OBED > OB > HC
Objective binge episodes	0.72 (3.14)	1.36 (2.45)	14.16 (21.46)	3.90*	OBED > HC/OB
Compensatory behaviour presence	7 (16.3)	9 (36.0)	9 (47.4)	7.12*	OBED > HC
Purging behaviour presence	0 (0.0%)	0 (0.0%)	2 (10.5%) ^a^	7.33*	OBED> HC/OB
Depression (DASS-21; 0–21)	2.09 (3.58)	1.96 (2.13)	6.79 (5.05)	7.74**	OBED > HC/OB
Grazing (GQ; 0–28)	7.35 (4.60)	11.52 (4.98)	15.74 (5.48)	20.21***	OBED > OB > HC
Grazing-repetitive eating (0–16)	5.19 (3.23)	7.04 (3.26)	8.58 (3.34)	7.68***	OBED > HC
Grazing-loss of control (0–12)	2.16 (2.10)	4.48 (2.29)	7.16 (2.43)	34.09***	OBED > OB > HC
Hunger (1–10)	4.33 (2.41)	4.56 (2.29)	5.05 (2.37)	0.62	–

Note: * *p* < .05. ** *p* < .01. *** *p* < .001 ^a^ The frequency of purging behaviour was lower than that specified in DSM-5 as criteria for purging bulimia nervosa

### Exclusions

One participant who endorsed significant ED psychopathology was excluded from the HC group. Two participants (one each from the HC and OB groups) were excluded as technical difficulties prevented instrumental task behavioural data from being recorded, and one additional participant from the OB group was excluded from ODT analyses only due to failure to acquire instrumental training. Therefore, demographic, clinical and CVT data is presented for 87 participants, and ODT data is presented for 86 participants.

### Clinical characteristics

The three groups differed significantly in terms of BMI, with the OBED group having the highest BMI, followed by the OB group, and then the HC group (group *p* < .001; Sidak *p* < .05 for pairwise comparisons). The groups also differed in terms of global ED psychopathology, depression severity, and severity of grazing, generally with OBED > OB > HC. The OBED group was the only one with an EDE-Q global score in the clinical range (*M*(*SD*) = 3.40 (0.72)); furthermore, all participants in this group endorsed objective binge episodes (*M*(*SD*) = 14.16 (21.46) episodes, range 1–84), with 63.2% of cases (*n* = 12) endorsing at least four such episodes over a four-week period, i.e. at least one such episode per week, on average. Almost half of the OBED group engaged in compensatory behaviour, most often driven exercise (36.8%), with only 10.5% endorsing purging. The OBED group can therefore be broadly characterised as exhibiting “binge eating disorder and non-purging bulimia nervosa spectrum” ED features. In terms of level of grazing, the HC group endorsed lower grazing severity than that reported by Lane and Szabó [36] in the GQ validation study which utilised a predominantly healthy-weight sample, while the grazing severity endorsed by the OBED group was comparable to that of a sub-threshold ED sample (but lower than that endorsed by a BN and binge eating disorder group) [53].

No significant differences between the groups existed in terms of current medication, alcohol consumption per week, smoking status, or hunger level at the time of the assessment.

### Action-outcome contingency sensitivity

#### Behavioural sensitivity

**Fig. 1:**
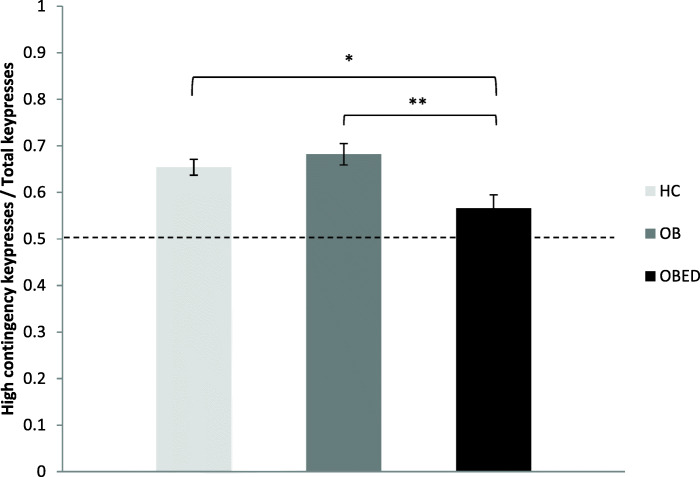
Behavioural sensitivity to contingency. * *p* < .05. ** *p* < .01

Although all three groups performed more than 50% of keypresses for the high contingency outcome, the OBED group performed a significantly lower number of presses for the high-contingency outcome as a proportion of total presses than both the HC group (*p* = .034) and the OB group (*p* = .005), demonstrating significantly lower action-outcome contingency sensitivity, while the OB and HC groups were not different to each other (*p* = .631); see Tables 2 and 3 and Fig. 1. When overall grazing severity, as well as “grazing: repetitive eating” severity and “grazing: loss of control” factor severity, were entered into analyses for contingency sensitivity, a differential pattern of results emerged for the three groups (Table 4). Grazing did not influence contingency sensitivity within the HC group or the OB group (all *p*s > .05). Within the OBED group, however, overall grazing severity (and that of the two subfactors) was associated with less diminished contingency sensitivity, such that for every point increase in grazing severity, contingency sensitivity increased by 2–5% (overall grazing severity: *p* < .001, “grazing: repetitive eating” severity: *p* < .001, GQ “grazing: loss of control” severity: *p* < .001).

**Table 2:** Unadjusted instrumental responding and ratings

	HC	OB	OBED	*F/Welch*	*p*	*ηp*^*2*^
	*M(SD)*	*M(SD)*	*M(SD)*			
	*n = 43*	*n = 25*	*n = 19*			
High contingency presses	153.00 (52.35)	146.37 (60.13)	115.04 (68.30)	2.86	.063	0.06
Low contingency presses	81.86 (34.88)	72.65 (41.37)	71.72 (35.49)	0.74	.481	0.02
CSI	0.65 (0.12)	0.67 (0.11)	0.59 (0.14)	2.64	.078	0.06
High contingency rating	5.38 (0.74)	5.44 (0.74)	5.48 (0.69)	0.11	.893	0.00
Low contingency rating	2.95 (0.87)	2.81 (0.96)	2.97 (0.70)	0.28	.760	0.01
	*n = 43*	*n = 24*	*n = 19*			
Nondevalued presses (training)	108.77 (53.13)	102.71 (55.78)	101.47 (45.16)	0.18	.840	0.00
Devalued presses (training)	105.42 (51.33)	101.13 (52.05)	91.95 (43.99)	0.48	.622	0.01
Nondevalued presses (extinction)	200.09 (95.82)	180.92 (86.37)	158.53 (90.85)	1.38	.257	0.03
Devalued presses (extinction)	85.26 (81.98)	90.33 (75.97)	62.74 (64.44)	0.78	.463	0.02
Nondevalued presses (reacquisition)	202.09 (98.26)	186.96 (95.66)	155.79 (96.87)	1.50	.230	0.04
Devalued presses (reacquisition)	82.37 (99.61)	70.79 (84.54)	47.63 (62.26)	1.43	.250	0.02
DSI (training)	0.51 (0.22)	0.50 (0.24)	0.52 (0.21)	0.05	.955	0.00
DSI (extinction)	0.70 (0.27)	0.67 (0.23)	0.73 (0.24)	0.28	.760	0.01
DSI (reacquisition)	0.73 (0.31)	0.73 (0.26)	0.77 (0.29)	0.13	.880	0.00
T2-T1 Nondevalued outcome pleasantness rating	0.17 (1.00)	0.06 (1.05)	0.16 (1.01)	1.00	.907	0.00
T2-T1 Devalued outcome pleasantness rating	−0.98 (1.88)	−1.04 (1.60)	−1.95 (2.01)	1.97	.146	0.05

**Table 3:** Instrumental responding and ratings adjusted for covariates

	HC	OB	OBED	*Wald χ*^*2*^_*(2)*_	*p*	Pairwise comparisons
	*EMM (SE)*	*EMM (SE)*	*EMM (SE)*			
	*n = 43*	*n = 25*	*n = 19*			
High contingency presses	155.42 (7.91)	146.88 (11.02)	105.54 (12.82)	8.21	.016	HC/OB > OBED
Low contingency presses	82.11 (5.17)	69.39 (7.12)	70.27 (6.19)	3.18	.204	–
CSI	0.65 (0.02)	0.68 (0.02)	0.57 (0.03)	9.32	.009	HC/OB > OBED
High contingency rating	5.32 (0.14)	5.43 (0.15)	5.63 (0.20)	1.50	.471	–
Low contingency rating	2.98 (0.13)	2.79 (0.18)	2.92 (0.15)	0.70	.705	–
	*n = 43*	*n = 24*	*n = 19*			
Nondevalued presses (training)	111.86 (17.80)	102.13 (21.30)	92.62 (23.88)	0.38	.827	–
Devalued presses (training)	98.47 (16.12	99.46 (21.48)	103.39 (29.85)	0.02	.991	–
Nondevalued presses (extinction)	201.45 (31.47)	178.93 (37.48)	154.20 (39.82)	0.78	.676	–
Devalued presses (extinction)	82.66 (14.10)	92.73 (20.03)	61.63 (17.65)	1.24	.537	–
Nondevalued presses (reacquisition)	205.84 (32.21)	185.13 (39.15)	146.71 (37.58)	1.20	.548	–
Devalued presses (reacquisition)	79.57 (13.79)	64.89 (13.70)	53.44 (16.58)	1.18	.556	–
DSI (training)	0.51 (0.04)	0.50 (0.05)	0.50 (0.06)	0.07	.967	–
DSI (extinction)	0.70 (0.06)	0.66 (0.08)	0.74 (0.11)	0.29	.864	–
DSI (reacquisition)	0.73 (0.06)	0.74 (0.08)	0.74 (0.10)	0.02	.991	–
T2-T1 Devalued outcome pleasantness rating	0.16 (0.15)	0.00 (0.21)	0.27 (0.26)	0.71	.700	–
T2-T1 Non-devalued outcome pleasantness rating	−1.00 (0.28)	−1.22 (0.37)	−1.67 (0.47)	1.42	.493	–

**Table 4:** Association between grazing and contingency sensitivity

	Grazing	HC (*n* = 43)		OB (*n* = 25)		OBED (*n* = 19)	
		*IRR [95% CI]*	*p*	*IRR [95% CI]*	*p*	*IRR [95% CI]*	*p*
CSI	Overall	1.00 [0.99, 1.01]	.587	1.00 [0.98, 1.02]	.980	1.02 [1.01, 1.03]	<.001
	Repetitive eating	1.00 [0.98, 1.01]	.498	1.01 [0.98, 1.03]	.740	1.04 [1.02, 1.06]	<.001
	Loss of control	1.00 [0.97, 1.02]	.927	0.99 [0.96, 1.03]	.731	1.05 [1.02, 1.07]	<.001

Note. IRRs are adjusted for covariates

#### Knowledge of contingency

**Fig. 2:**
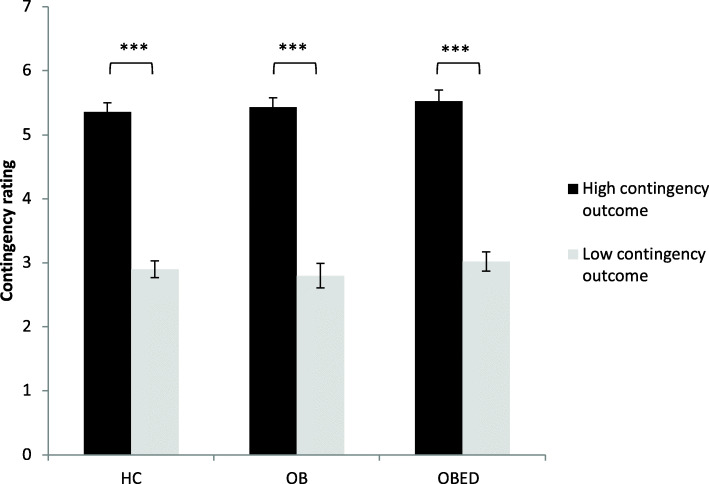
Ratings of action-outcome contingency. *** *p* < .001

There were no group-based differences in terms of contingency ratings; all groups correctly rated presses for the high contingency as being substantially more effective in obtaining this outcome than presses for the low contingency outcome were for obtaining this outcome (group *p* = .649; contingency *p* < .001; group x contingency *p* = .800); see Tables 2 and 3 and Fig. 2.

### Outcome value sensitivity

#### Behavioural sensitivity

**Fig. 3:**
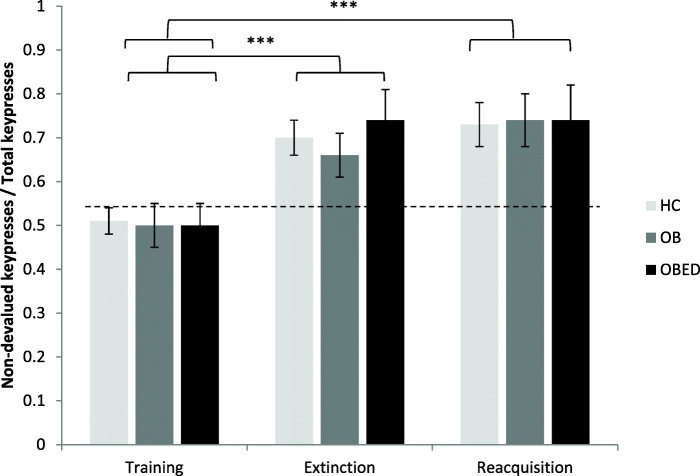
Behavioural sensitivity to outcome devaluation. *** *p* < .001

All participants performed a significantly higher number of presses for the nondevalued outcome as a proportion of total presses in both the extinction and the reacquisition stage, compared to the instrumental training stage, without any significant between-group differences found (extinction: group *p* = .859, stage *p* < .001, group x stage *p* = .942; reacquisition: group *p* = .994, stage *p* < .001, group x stage *p* = .994); see Tables 2 and 3 and Fig. 3.

#### Change in pleasantness ratings

**Fig. 4:**
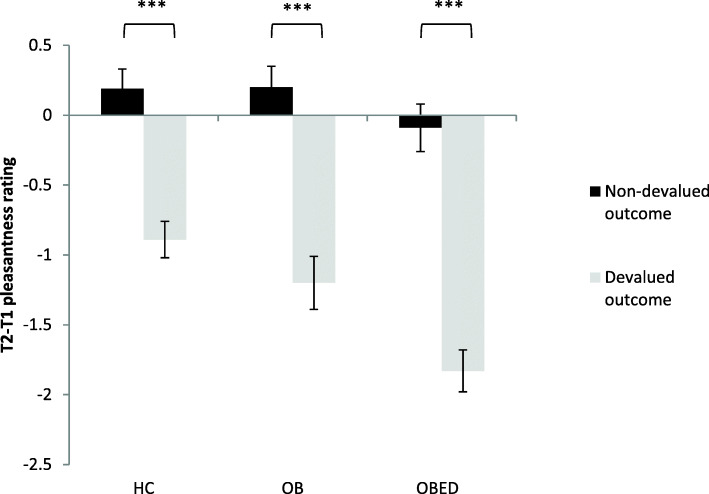
Changes in pleasantness rating between T1 and T2. *** *p* < .001

There were no group-based differences in terms of change in pleasantness ratings; all three groups displayed a strong subjective devaluation effect, displaying a large drop in pleasantness between T1 and T2 for the devalued outcome, but not for the nondevalued outcome (group *p* = .177; devaluation *p* < .001; group x devaluation *p* = .418); see Tables 2 and 3 and Fig. 4.

## Discussion

The present study is the first to relate grazing to the basis of instrumental decision-making; it aimed to examine differences across two aspects of goal-directed behaviour (contingency sensitivity and devaluation sensitivity) between a group with obesity with and without significant ED features and a healthy control group, and to relate any differences in goal-directed behaviour to grazing severity within these groups. All three study groups demonstrated both behavioural and subjective sensitivity to outcome devaluation. However, while all three groups demonstrated intact action-outcome contingency knowledge, OBED participants displayed a reduced behavioural sensitivity to contingency compared to both OB and HC participants. The observed reduced sensitivity to contingency indicates that OBED participants were not able to flexibly shift their behaviour towards the more advantageous action based on feedback received despite intact knowledge of contingency, indicating a more habitual response style. This reduced flexibility to changes in action/outcome relationships could be related to sub-optimal decision-making, especially in food-related contexts, and could therefore represent a driver of compulsive eating behaviour that should be considered in treatment. Behaviour which is more reflexive and habitual is more resistant to modification, hence treatment should incorporate ways to restore goal-directedness to unhelpful behaviour, potentially by incorporating strategies from the habit reversal clinical literature. Inaccurate mapping of causal knowledge onto behaviour could also reduce the initiation of optimal health behaviour actions, which has additional clinical implications in groups with both ED psychopathology and high weight, where such actions may be specifically targeted in treatment (e.g. [54]).

There is some indication that habit does not always moderate the intention-behaviour gap when the intention is to inhibit an undesired behaviour (e.g. to stop snacking on unhealthy food), rather than to perform a desired behaviour (e.g. to snack on healthy food) [55], however this relationship itself may be moderated by an ability to self-control [56]. Given that the OBED group was defined predominantly by binge-type ED behaviours, characterised by a sense of loss of control over eating, it is not surprising that when food cues were presented as part of the tasks in the current study, those with obesity combined with ED features were less able to maintain goal-directed responding than those with obesity without ED features (who may have more self-control resources at their disposal when cued with food), as well as HC participants. This contributes to a growing body of literature indicating that in persons with ED, including those with obesity, selective cognitive processing biases exist when disorder-specific stimuli (i.e. related to food/eating, or weight/shape) are present [57, 58]. In the current study, OB participants performed similarly to HC participants, suggesting that a combination of high weight and ED psychopathology may incline behaviour towards cue-driven habits, rather than high weight alone. It is also possible that existing alterations in frontostriatal networks, reflected in less goal-directed behaviour, may be a predisposing factor for both obesity and ED psychopathology [59].

The finding of retained sensitivity to outcome value in the OB and OBED groups is at odds with some recent findings [17, 18]. One difference between previous work and the current study is the method used to devalue an outcome; whereas previous studies have largely used selective satiation [17, 18], allowing participants to freely consume one of the trained outcomes, here devaluation was achieved by having participants view a video of cockroaches making contact with one of the trained outcomes, presumably evoking a disgust reaction. It is possible that this is a stronger means of devaluation that overcomes deficits that may be detected with outcome-specific satiety. Further, is likely that satiety mechanisms differ in obesity and so possible that previously reported deficits relate, in part, to the efficacy of the devaluation treatment in addition to differences in goal-directed control. These types of procedural differences make comparisons across studies complex and highlight a need to validate and standardise outcome devaluation paradigms. The sensitivity of responding to devaluation indicates that all groups learned the initial action-outcome relationships. Nonetheless, the reduced sensitivity to contingency changes reveals difficulty updating previous learning and indicates reduced flexible control over established responses that could present a challenge for behaviour change.

Interestingly, grazing severity appeared to have some associations with goal-directedness but only in the OBED group, where it was associated with less diminished action-outcome contingency. It is possible that within this group grazing *does* represent a type of goal-directed eating behaviour more akin to planned snacking [35], i.e. for those wishing to inhibit the consumption of larger amounts of food such as those characteristic of objective binge episodes or objective overeating episodes, behaviour may be redirected towards the consumption of smaller amounts of food. This is also consistent with findings that within a community sample meeting DSM-5 criteria for bulimia nervosa, healthy-weight individuals grazed more than overweight individuals, with the opposite pattern observed for individuals meeting binge eating disorder criteria [53]. This suggests that grazing could in some circumstances be deployed as a compensatory or substitutive behaviour. It is possible that a relatively higher ability for self-control creates an interface between dietary intentions, habit strength, and eating behaviour, such that in those wishing not to consume large amounts of food, even where such consumption patterns are habitual, inhibitory control may allow the redirection of eating behaviour towards grazing. Whether inhibitory control mediates the relationship between goal-direction/habit and eating behaviour, or conversely, goal-direction/habit mediates the relationship between inhibition and eating behaviour represents an area of future research. The amount of food consumed through grazing over a long time period of time may still be substantial, and may thus contribute to weight gain or to maintenance of a higher weight [32, 38]; furthermore, detrimental associations have been found between grazing and mental health, mental health-related quality of life [32, 38] and grazing-related distress, hence this compensatory/substitutive strategy may not be effective or helpful in the long term.

It is also possible that the type of grazing experienced by the current study sample was not very severe or compulsive, as evidenced by relatively low mean scores for the GQ and its two factors (“repetitive eating” and “loss of control”) even in the group with obesity and ED features. For some post-bariatric surgery patients, grazing was seen as an adaptive form of eating, representing mindful and healthy food choices taken in small amounts throughout the day, reflecting a low level of compulsiveness [60]; hence there is a possibility that while lower-severity grazing is associated with more adaptive decision-making, as severity increases, especially for its more compulsive element, the relationship with decision-making may become less positive. Measuring compulsive and non-compulsive grazing frequency across a defined time period as another index of severity could be beneficial in future research, e.g. using different instruments such as the Short Inventory of Grazing [45].

One potential reason for the lack of significant differences between HC and OB participants may be that the participants with obesity in the current study tended to be young, well-educated and relatively healthy (as suggested by the lack of endorsed chronic diseases, and very low rates of blood pressure and cholesterol medication use, tobacco, alcohol and other substance use, and endorsement of weekly exercise). It is possible that effects of obesity on cognition and decision-making manifest in later age; for example, a recent meta-analysis found these effects in adult, but not adolescent samples [27]. Alternatively, that there may be a mediating effect of obesity-related conditions such as hypertension and insulin resistance between body weight and cognitive performance [61, 62].

The current study has several strengths; it is one of the first studies to look at both contingency sensitivity and outcome value sensitivity within the context of obesity and ED within a human sample, and the first to relate goal-directed behaviour to grazing. The study sample included both men and women who were assessed for potential confounds such as metabolic conditions and substance use; groups were matched through sampling and/or controlled statistically for important variables such as sex, age, education, socioeconomic status, overall intellectual functioning, depression severity, and baseline hunger level. Outcomes in the current study were food pictures, which possess increased relevance compared to abstract stimuli or monetary rewards, and have been found to have a similar effect as real food exposure in predicting food-related outcomes [63]. Some limitations were also present; although the sample size used was adequate for detecting large effect sizes, a larger sample would have been necessary to detect more subtle differences between the groups; additionally, the inclusion of a subgroup of healthy-weight participants with ED symptomatology would facilitate separating the contributions of high weight and psychopathology to reductions in goal-directed behaviour. The norms used to categorise participants as likely to meet criteria for an ED were derived from community samples, whereas a small number of participants in the current sample (9%) were recruited from a university setting. Rewards in this study were presented through passive viewing, rather than actual consumption of food items, and a disgust devaluation procedure was used rather than the more commonly-used devaluation by satiety procedure; it is possible that pictorial representations of snack food had reduced behavioural relevance and motivational effects [20], leading to an overall reduction in differential responding (e.g. see Medic, Ziauddeen [64]). However, overall, participants did perform more presses for the high-contingency outcome in the CVT and for the nondevalued outcome following devaluation in the ODT, indicating that the current experimental procedure was effective.

Future research should seek to incorporate other cognitive elements that may influence the relationship between high weight and goal-directed behaviour, such as inhibition, cognitive restraint [65], impulsivity, cognitive load, reward sensitivity; it should consider the role of inflammatory markers and/or appetite-regulating hormones [66]; and it should use in vivo tasks, e.g. experimental paradigms using actual food, or using ecological momentary assessment methods. Neuroimaging studies examining neural functioning in persons who engage in more severe grazing could also be conducted, and comparisons made with persons who engage in other forms of atypical eating. The link between grazing and other eating patterns on the “compulsive eating” spectrum (such as binge eating) should be further investigated and clarified, especially given shared components. Severity and duration of obesity should also be taken into account [66], and overweight groups should also be included (or BMI included as a continuous predictor), to examine potential graded effects of weight; other measures of adiposity (such as waist circumference) should also be considered in addition to BMI, or composite indices (e.g. Janssen et al. [17]). The construct of grazing itself could be further refined by investigating types and amount of food typically consumed, length and timing of grazing periods (especially in light of emerging findings regarding potential metabolic benefits of early time-restricted feeding [67, 68]); additionally, using measures of grazing which better discriminate between grazing which is lower or higher in compulsivity such as the Rep(eat)-Q [38] or the SIG [45] would be indicated for a finer-grained analysis of the relationships between different aspects of grazing and cognitive processing, i.e. if higher compulsivity or higher repetitiveness displays a less favourable pattern of associations; it was not possible to determine this in the current study, as the two factors of the GQ were highly and positively correlated. More broadly, longitudinal studies are needed to investigate how specific factors (e.g. increasing weight or grazing frequency) may relate to neurobiological features, and to answer questions of causality, such as whether less goal-directed behaviour is a risk factor for developing higher weight via unhelpful eating behaviours, or whether higher weight leads to a vulnerability in terms of accelerated transition to habitual behavioural control.

## Conclusions

The current study investigated goal-directed behaviour and its association with grazing in a group of participants with obesity with and without ED features, compared to a healthy control group. Lower action-outcome contingency sensitivity was found in the group with obesity and with ED features than in the other groups, despite intact explicit knowledge of contingency, indicating less goal-directed behaviour in this group. This impairment in contingency-related behavioural flexibility may represent one of the drivers of compulsive eating behaviour; treatment approaches should seek to use explicit knowledge of the contingencies between actions and outcomes to update behaviour. A small but significant positive relationship was found between behavioural contingency sensitivity and grazing in the group with obesity and ED features, suggesting that in this group grazing may represent a more goal-directed behaviour, potentially deployed as a compensatory or substitutive strategy, despite potentially unhelpful long-term implications of grazing.

## Supplementary information

**Additional file 1.** Additional methods: instrumental decision-making task procedure and pleasantness rating scale; Microsoft Word document.

## Data Availability

The datasets used and/or analysed during the current study are available from the corresponding author on reasonable request.

## Abbreviations

BMI: Body mass index
CSI: Contingency Sensitivity Index
CVT: Contingency Variation Task
DASS-21: Depression Anxiety Stress Scales-21
DSI: Devaluation Sensitivity Index
ED: Eating disorder
EDE-Q: Eating Disorder Examination-Questionnaire
GQ: Grazing Questionnaire
HC: Healthy control group
IRR: Incidence risk ratio
OB: Group with obesity and without significant ED features
OBED: Group with obesity and with significant ED features
ODT: Outcome Devaluation Task
TOPF: Test of Premorbid Functioning

## References

[1] Wang YC, McPherson K, Marsh T, Gortmaker SL, Brown M. Health and economic burden of the projected obesity trends in the USA and the UK. Lancet. 2011;378(9793):815–25. 10.1016/S0140-6736(11)60814-3 21872750

[2] National Health and Medical Research Council (NHMRC). Clinical practice guidelines for the management of overweight and obesity in adults, adolescents and children in Australia. Melbourne: National Health and Medical Research Council. 2013. https://www.nhmrc.gov.au/about-us/publications/clinical-practice-guidelines-management-overweight-and-obesity. Accessed 14 Sept 2020.

[3] Palavras MA, Kaio GH, Mari JdJ, Claudino AM. A review of Latin American studies on binge eating disorder. Brazilian Journal of Psychiatry. 2019;33:s81–s94. 10.1590/s1516-44462011000500007 21845337

[4] Udo T, Grilo CM. Prevalence and correlates of DSM-5-defined eating disorders in a nationally representative sample of US adults. Biol Psychiatry. 2018;84(5):345–54. 10.1016/j.biopsych.2018.03.014 PMC6097933 29859631

[5] da Luz FQ, Hay P, Touyz S, Sainsbury A. Obesity with comorbid eating disorders: Associated health risks and treatment approaches. Nutrients. 2018;10(7):829. 10.3390/nu10070829 PMC6073367 29954056

[6] da Luz FQ, Sainsbury A, Touyz S, Mannan H, Hay P, Mitchison D. Prevalence of obesity and comorbid eating disorder behaviors in South Australia from 1995 to 2015. Int J Obes. 2017;41(7):1148–53. 10.1038/ijo.2017.79 28337025

[7] Aarts H, Paulussen T, Schaalma H (1997). Physical exercise habit: on the conceptualization and formation of habitual health behaviours. Health Educ Res.

[8] van't Riet J, Sijtsema SJ, Dagevos H, De Bruijn GJ (2011). The importance of habits in eating behaviour: an overview and recommendations for future research. Appetite..

[9] Skinner BF (1938). The behavior of organisms: an experimental analysis.

[10] Dickinson A (1985). Actions and habits: the development of behavioural autonomy. Philos Trans R Soc Lond B Biol Sci.

[11] Griffiths KR, Morris RW, Balleine BW (2014). Translational studies of goal-directed action as a framework for classifying deficits across psychiatric disorders. Front Syst Neurosci.

[12] Killcross S, Coutureau E (2003). Coordination of actions and habits in the medial prefrontal cortex of rats. Cereb Cortex.

[13] Nordquist RE, Voorn P, de Mooij-van Malsen JG, Joosten RN, Pennartz CM, Vanderschuren LJ (2007). Augmented reinforcer value and accelerated habit formation after repeated amphetamine treatment. Eur Neuropsychopharmacol.

[14] Schneider W, Chein JM (2003). Controlled & automatic processing: behavior, theory, and biological mechanisms. Cogn Sci.

[15] Adams CD (1982). Variations in the sensitivity of instrumental responding to reinforcer devaluation. Q J Exp Psychol B.

[16] Balleine BW, Dickinson A (1991). Instrumental performance following reinforcer devaluation depends upon incentive learning. Q J Exp Psychol B.

[17] Janssen LK, Duif I, van Loon I, Wegman J, de Vries JHM, Cools R (2017). Loss of lateral prefrontal cortex control in food-directed attention and goal-directed food choice in obesity. NeuroImage..

[18] Horstmann A, Dietrich A, Mathar D, Possel M, Villringer A, Neumann J (2015). Slave to habit? Obesity is associated with decreased behavioural sensitivity to reward devaluation. Appetite..

[19] Voon V, Derbyshire K, Ruck C, Irvine MA, Worbe Y, Enander J (2015). Disorders of compulsivity: a common bias towards learning habits. Mol Psychiatry.

[20] Schienle A, Schäfer A, Hermann A, Vaitl D (2009). Binge-eating disorder: reward sensitivity and brain activation to images of food. Biol Psychiatry.

[21] Goodnight J, Bornstein M (2018). Reward sensitivity. The SAGE encyclopedia of lifespan human development.

[22] Veldhoven DT-v, Roozen H, Vingerhoets A (2020). The association between reward sensitivity and activity engagement: the influence of delay discounting and anhedonia. Alcohol Alcohol.

[23] Everitt BJ, Robbins TW (2005). Neural systems of reinforcement for drug addiction: from actions to habits to compulsion. Nat Neurosci.

[24] Corbit LH (2016). Effects of obesogenic diets on learning and habitual responding. Curr Opin Behav Sci.

[25] Tricomi E, Balleine BW, O'Doherty JP (2009). A specific role for posterior dorsolateral striatum in human habit learning. Eur J Neurosci.

[26] Wang GJ, Geliebter A, Volkow ND, Telang FW, Logan J, Jayne MC (2011). Enhanced striatal dopamine release during food stimulation in binge eating disorder. Obesity..

[27] Wu M, Brockmeyer T, Hartmann M, Skunde M, Herzog W, Friederich H-C (2016). Reward-related decision making in eating and weight disorders: a systematic review and meta-analysis of the evidence from neuropsychological studies. Neurosci Biobehav Rev.

[28] Carter FA, Jansen A (2012). Improving psychological treatment for obesity. Which eating behaviours should we target?. Appetite..

[29] Davis C. A commentary on the associations among ‘food addiction’, binge eating disorder, and obesity: overlapping conditions with idiosyncratic clinical features. Appetite. 2017;115:3–8. 10.1016/j.appet.2016.11.001. 10.1016/j.appet.2016.11.001 27816464

[30] Saunders R, Johnson L, Teschner J (1998). Prevalence of eating disorders among bariatric surgery patients. Eat Disord.

[31] Heriseanu AI, Hay P, Corbit L, Touyz S (2017). Grazing in adults with obesity and eating disorders: a systematic review of associated clinical features and meta-analysis of prevalence. Clin Psychol Rev.

[32] Heriseanu AI, Hay P, Touyz S (2019). Grazing behaviour and associations with obesity, eating disorders, and health-related quality of life in the Australian population. Appetite.

[33] Conceição EM, Mitchell JE, Engel S, Machado P, Lancaster K, Wonderlich S (2014). What is "grazing"? Reviewing its definition, frequency, clinical characteristics, and impact on bariatric surgery outcomes, and proposing a standardized definition. Surg Obes Relat Dis.

[34] Conceição EM, de Lourdes M, Pinto-Bastos A, Vaz AR, Brandão I, Ramalho S (2018). Problematic eating behaviors and psychopathology in patients undergoing bariatric surgery: the mediating role of loss of control eating. Int J Eat Disord.

[35] Fairburn CG (2008). Cognitive behaviour therapy and eating disorders.

[36] Lane B, Szabó M (2013). Uncontrolled, repetitive eating of small amounts of food or 'grazing': development and evaluation of a new measure of atypical eating. Behav Chang.

[37] Colles SL, Dixon JB, O'Brien PE (2008). Grazing and loss of control related to eating: two high-risk factors following bariatric surgery. Obesity..

[38] Conceição EM, Mitchell JE, Machado PPP, Vaz AR, Pinto-Bastos A, Ramalho S (2017). Repetitive eating questionnaire [rep(eat)-Q]: enlightening the concept of grazing and psychometric properties in a Portuguese sample. Appetite..

[39] Bonder R, Davis C, Kuk JL, Loxton NJ (2018). Compulsive “grazing” and addictive tendencies towards food. Eur Eat Disord Rev.

[40] Raman J, Smith E, Hay P (2013). The Clinical Obesity Maintenance Model: An integration of psychological constructs including mood, emotional regulation, disordered overeating. habitual cluster behaviours, health literacy and cognitive function. J Obes.

[41] Allom V, Mullan B, Smith E, Hay P, Raman J (2018). Breaking bad habits by improving executive function in individuals with obesity. BMC Public Health.

[42] Fairburn CG, Beglin SJ (1994). Assessment of eating disorder psychopathology: interview or self-report questionnaire?. Int J Eat Disord.

[43] Burton AL, Abbott MJ, Modini M, Touyz S (2016). Psychometric evaluation of self-report measures of binge-eating symptoms and related psychopathology: a systematic review of the literature. Int J Eat Disord.

[44] Mond JM, Hay P, Rodgers B, Owen C, Beumont PJV (2004). Validity of the eating disorder examination questionnaire (EDE-Q) in screening for eating disorders in community samples. Behav Res Ther.

[45] Heriseanu AI, Hay P, Touyz S. The Short Inventory of Grazing (SIG): Development and validation of a new brief measure of a common eating behaviour with a compulsive dimension. J Eat Disord. 2019;7(4). 10.1186/s40337-019-0234-6. 10.1186/s40337-019-0234-6 PMC6366119 30774954

[46] Balleine BW, O'Doherty JP (2010). Human and rodent homologies in action control: corticostriatal determinants of goal-directed and habitual action. Neuropsychopharmacology..

[47] Peirce JW. Generating stimuli for neuroscience using PsychoPy. Front Neuroinformatics. 2009; 2(10). 10.3389/neuro.11.010.2008. 10.3389/neuro.11.010.2008 PMC2636899 19198666

[48] Dietrich A, de Wit S, Horstmann A. General habit propensity relates to the sensation seeking subdomain of impulsivity but not obesity. Front Behav Neurosci. 2016;10(213). 10.3389/fnbeh.2016.00213. 10.3389/fnbeh.2016.00213 PMC5099246 27877117

[49] Lovibond SH, Lovibond PF (1995). Manual for the depression anxiety stress scales 2nd ed.

[50] Antony MM, Bieling PJ, Cox BJ, Enns MW, Swinson RP (1998). Psychometric properties of the 42-item and 21-item versions of the depression anxiety stress scales (DASS) in clinical groups and a community sample. Psychol Assess.

[51] Wechsler D (2009). Test of premorbid functioning.

[52] Prickett C, Brennan L, Stolwyk R (2015). Examining the relationship between obesity and cognitive function: a systematic literature review. Obes Res Clin Pract.

[53] Holzner L, Szabo M (2014). Uncontrolled, repetitive eating of small amounts of food or 'grazing': Initial assessment in a community sample of binge eaters.

[54] da Luz FQ, Swinbourne J, Sainsbury A, Touyz S, Palavras M, Claudino A (2017). HAPIFED: a healthy APproach to weIght management and food in eating disorders: a case series and manual development. J Eat Disord.

[55] Gardner B, Corbridge S, McGowan L (2015). Do habits always override intentions? Pitting unhealthy snacking habits against snack-avoidance intentions. BMC Psychology.

[56] Neal D, Wood W, Drolet A (2013). How do people adhere to goals when willpower is low? The profits (and pitfalls) of strong habits. J Pers Soc Psychol.

[57] Van den Eynde F, Guillaume S, Broadbent H, Stahl D, Campbell IC, Schmidt U (2011). Neurocognition in bulimic eating disorders: a systematic review. Acta Psychiatr Scand.

[58] Kittel R, Brauhardt A, Hilbert A (2015). Cognitive and emotional functioning in binge-eating disorder: a systematic review. Int J Eat Disord.

[59] Michaelides M, Thanos PK, Volkow ND, Wang G-J (2012). Dopamine-related frontostriatal abnormalities in obesity and binge-eating disorder: emerging evidence for developmental psychopathology. Int Rev Psychiatry.

[60] Zunker C, Karr T, Saunders R, Mitchell JE (2012). Eating behaviors post-bariatric surgery: a qualitative study of grazing. Obes Surg.

[61] Gonzales MM, Tarumi T, Miles SC, Tanaka H, Shah F, Haley AP (2010). Insulin sensitivity as a mediator of the relationship between BMI and working memory-related brain activation. Obesity..

[62] Elias MF, Elias PK, Sullivan LM, Wolf PA, D'Agostino RB (2003). Lower cognitive function in the presence of obesity and hypertension: the Framingham heart study. Int J Obes.

[63] Boswell RG, Kober H (2016). Food cue reactivity and craving predict eating and weight gain: a meta-analytic review. Obes Rev.

[64] Medic N, Ziauddeen H, Forwood SE, Davies KM, Ahern AL, Jebb SA, et al. The presence of real food usurps hypothetical health value judgment in overweight people. eNeuro. 2016;3(2). 10.1523/eneuro.0025-16.2016. 10.1523/ENEURO.0025-16.2016 PMC4894914 27280152

[65] Preuss H, Leister L, Pinnow M, Legenbauer T. Inhibitory control pathway to disinhibited eating: a matter of perspective? Appetite. 2019;141. 10.1016/j.appet.2019.05.028 31128199

[66] Janssen LK, Herzog N, Waltmann M, Breuer N, Wiencke K, Rausch F, et al. Lost in translation? On the need for convergence in animal and human studies on the role of dopamine in diet-induced obesity. Curr Addict Rep. 2019;6:229–57. 10.1007/s40429-019-00268-w.

[67] Chaix A, Lin T, Le HD, Chang MW, Panda S (2019). Time-restricted feeding prevents obesity and metabolic syndrome in mice lacking a circadian clock. Cell Metab.

[68] Jamshed H, Beyl RA, Della Manna DL, Yang ES, Ravussin E, Peterson CM (2019). Early time-restricted feeding improves 24-hour glucose levels and affects markers of the circadian clock, aging, and autophagy in humans. Nutrients..

