# Supplementary material for: Relating goal-directed behaviour to grazing in persons with obesity with and without eating disorder features
Source: J Eat Disord. 2020 Oct 1;8:48. doi: 10.1186/s40337-020-00324-1 (PMC7528325; doi:10.1186/s40337-020-00324-1)

**Additional File 1 - Instrumental task procedure**

**Contingency Variation Task (CVT)**

Participants received the following instructions:

"*Someone has told you that free snacks can be won from our vending machines by tilting them to the left or to the right. Use the Left and Right keys to tilt left and right. Find out which direction releases the most snacks, and try to win as many snacks as you can!*" Following these instruction, the CVT consisted of two phases, which were repeated over six blocks:

1. Keypresses to earn reinforcers: Across six blocks, pressing "left" (L) and "right" (R) on a "vending machine" led to earning two different snacks (snacks A and B, presented as pictures). Within each block, each snack was rewarded on either a high, or a low action-outcome contingency. On average one in four (high contingency), or one in 12 (low contingency) keypresses were followed by the delivery of a reward). Participant L / R responses were recorded. The duration for each block was 60 seconds. Snack allocation to L vs. R keypresses, and to high vs. low contingency, was counterbalanced across blocks. The mean number of keypresses for high contingency (HC), and for low contingency (LC) outcomes across the six blocks were used as the measure of behavioural contingency sensitivity.
2. Rating of contingency: At the conclusion of each block, knowledge of contingency was rated: participants were asked to rate how likely the vending machine was to deliver each of the two snacks: "*How effective was tilting the machine to get Snack A?*" and "*How effective was tilting the machine to get Snack B?*" These were rated on a scale of 1 to 7, with 1 representing "not at all effective" and 7 representing "very effective". The average rating for the HC outcome and for the LC outcome constituted the measure of explicit knowledge of contingency.

**Outcome Devaluation Task (ODT)**

The ODT consisted of four phases:

1. Instrumental training. In the initial training phase, participants received the following instructions: "*You can win snacks from our vending machine by tilting it to the left or to the right using the keyboard keys. If you tilt the machine one way you can win one kind of snack, and if you tilt it the other way you can win a different kind of snack. Press the buttons to learn what snack you can win by tilting in each direction, and try to win as many snacks as you can!*" Participants learned the association between their responses (L or R) on two keyboard keys and the possibility of winning one of two rewards (pictures of snacks A or B); L and R presses earned a different snack food, and this was counterbalanced between participants. The training phase was not time-limited, and was complete once participants had won a total of 25 rewards. Both the L and R rewards were delivered on a RR (random ratio) 5 reinforcement schedule, where there is a 0.2 probability of reinforcement being delivered after each response; therefore, a high response rate increased the possibility of winning a higher quantity of food rewards. Feedback (number of each of the two snacks won) was given to participants at the completion of the training phase. Number of responses for the two snacks was recorded.
2. Outcome devaluation. Once training was complete, participants were told that: "*Something has happened to one of the snacks! Watch now to see what happened.*" This was followed by a 90-second video showing one of the snack foods (A or B, counterbalanced) contaminated by cockroaches.
3. Extinction test. Participants were then told: "*You can now try to win snacks from the vending machine by tilting it, however now you will not be able to see the snacks appear as you win them. Try to win as many snacks as you can!*" The virtual vending machine was displayed again for 60 seconds, during which time participants could tilt the machine L or R at will, in the absence of pictures of snack food.
4. Instrumental reacquisition. Following testing under extinction conditions, participants were instructed: "*Again, you can tilt the vending machine to win snacks. You will now be able to see the snacks as you win them. Try to win as many snacks as you can!*" and responses were once again reinforced with pictures of snacks A and B on a RR5 schedule, for 60 seconds. Feedback (number of snack A and B won) was given at the completion of this phase.

**Pictorial representations**

Figure 1. Pictures of snacks used as outcomes.


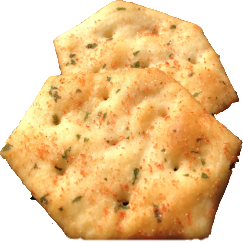

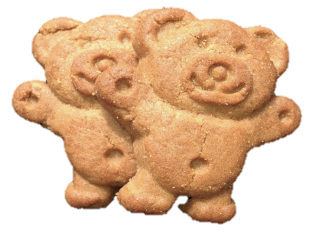

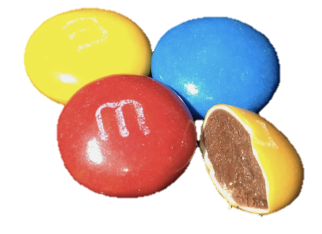


M&Ms BBQ Shapes Tiny Teddies

Figure 2. Representation of the "vending machine".


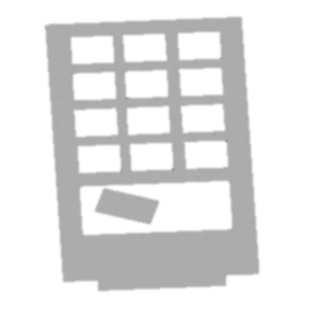

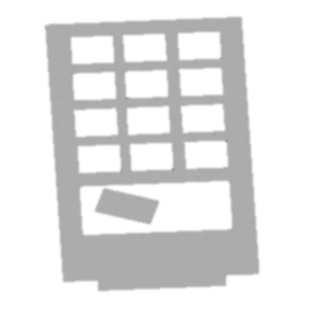


L - tilting left R - tilting right


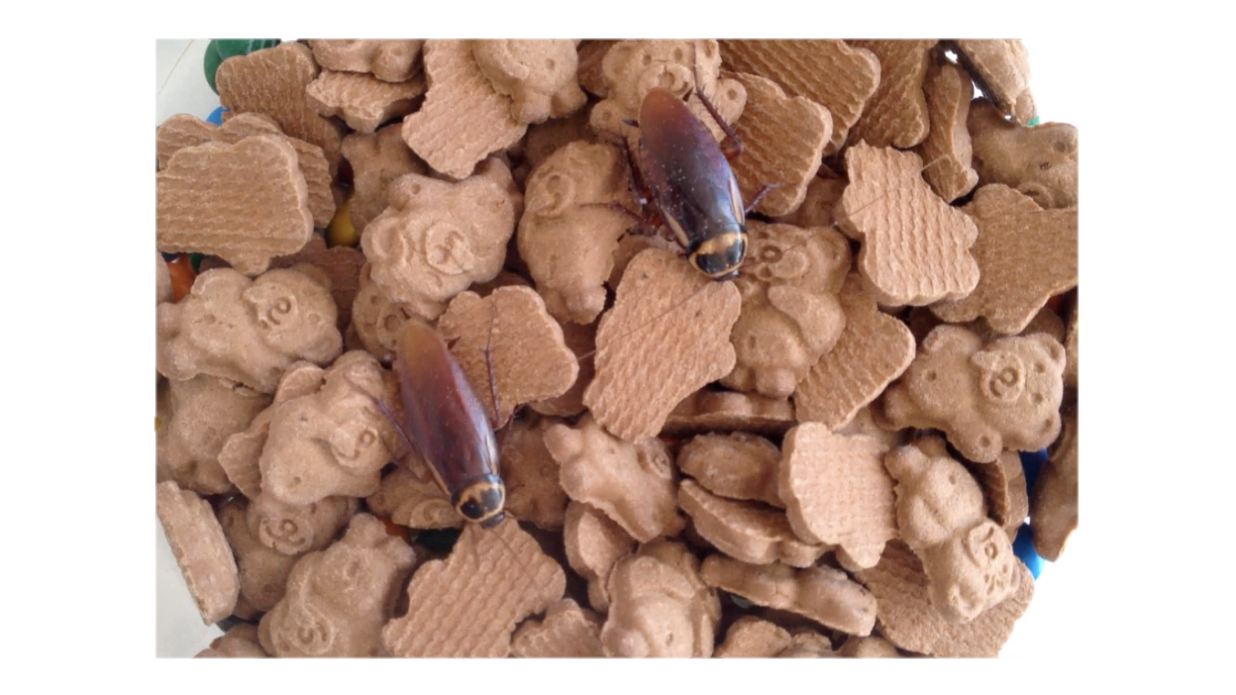
Figure 3. Still from a devaluation procedure video showing the devaluation of one of the study snacks (Tiny Teddies).


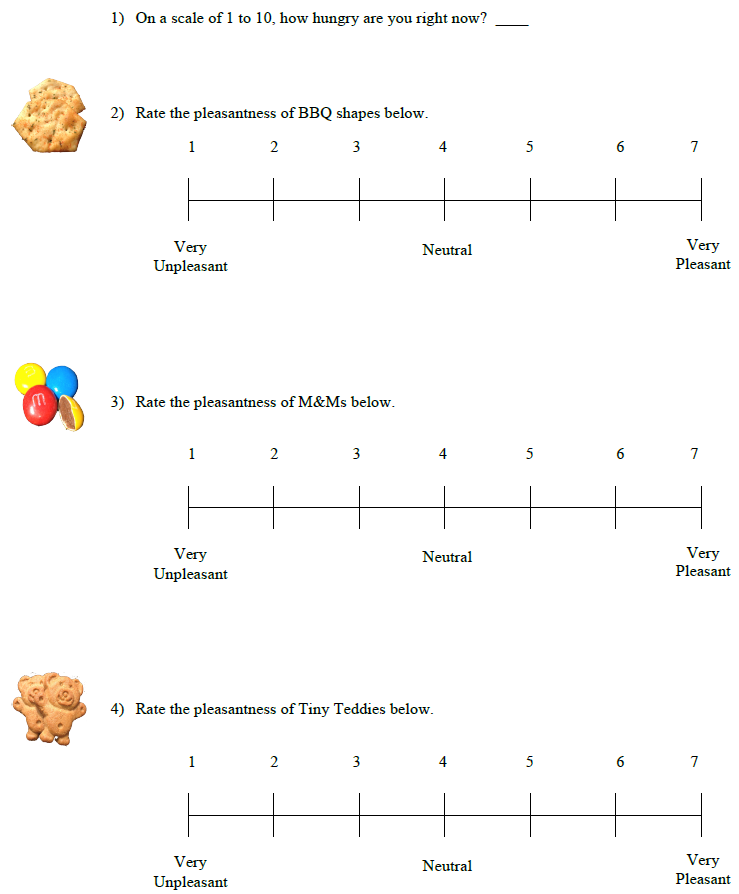

Supplement: Supplementary file 1 — Additional file 1. Additional methods: instrumental decision-making task procedure and pleasantness rating scale; Microsoft Word document. [file 40337_2020_324_MOESM1_ESM.docx]
